# Automated exploitation of deep learning for cancer patient stratification across multiple types

**DOI:** 10.1093/bioinformatics/btad654

**Published:** 2023-11-02

**Authors:** Pingping Sun, Shijie Fan, Shaochuan Li, Yingwei Zhao, Chang Lu, Ka-Chun Wong, Xiangtao Li

**Affiliations:** School of Information Science and Technology, Northeast Normal University, Jilin, China; School of Information Science and Technology, Northeast Normal University, Jilin, China; School of Information Science and Technology, Northeast Normal University, Jilin, China; School of Artificial Intelligence, Jilin University, Jilin, China; School of Information Science and Technology, Northeast Normal University, Jilin, China; School of Information Science and Technology, Northeast Normal University, Jilin, China; School of Psychology, Northeast Normal University, Jilin, China; Department of Computer Science, City University of Hong Kong, Hong Kong China; School of Artificial Intelligence, Jilin University, Jilin, China

## Abstract

**Motivation:**

Recent frameworks based on deep learning have been developed to identify cancer subtypes from high-throughput gene expression profiles. Unfortunately, the performance of deep learning is highly dependent on its neural network architectures which are often hand-crafted with expertise in deep neural networks, meanwhile, the optimization and adjustment of the network are usually costly and time consuming.

**Results:**

To address such limitations, we proposed a fully automated deep neural architecture search model for diagnosing consensus molecular subtypes from gene expression data (DNAS). The proposed model uses ant colony algorithm, one of the heuristic swarm intelligence algorithms, to search and optimize neural network architecture, and it can automatically find the optimal deep learning model architecture for cancer diagnosis in its search space. We validated DNAS on eight colorectal cancer datasets, achieving the average accuracy of 95.48%, the average specificity of 98.07%, and the average sensitivity of 96.24%, respectively. Without the loss of generality, we investigated the general applicability of DNAS further on other cancer types from different platforms including lung cancer and breast cancer, and DNAS achieved an area under the curve of 95% and 96%, respectively. In addition, we conducted gene ontology enrichment and pathological analysis to reveal interesting insights into cancer subtype identification and characterization across multiple cancer types.

**Availability and implementation:**

The source code and data can be downloaded from https://github.com/userd113/DNAS-main. And the web server of DNAS is publicly accessible at 119.45.145.120:5001.

## 1 Introduction

Colorectal cancer (CRC) is a very heterogeneous disease and one of the leading causes of cancer-related deaths worldwide (Biller and Schrag 2021). Unfortunately, the number of deaths is anticipated to keep rising even in developed countries (Rahib *et al.* 2014). Until recently, most CRC studies have focused on histopathological classification, single-molecular condition identification such as microsatellite instability (MSI) or different mutation status of major cancer genes (Fessler *et al.* 2016). Nevertheless, due to the diversity and complexity of CRC, the precise diagnosis and treatment of CRC patients poses a huge challenge.

Recently, a consensus molecular subtype-based classification has demonstrated potential as a framework to provide subtype-specific directed therapy of colorectal cancer in the clinical setting (Singh *et al.* 2021). Indeed, the CRC Subtyping Consortium (CRCSC) collected thousands of samples of four consensus molecular subtypes (CMSs) (Guinney *et al.* 2015): CMS1 (microsatellite instability immune), CMS2 (canonical), CMS3 (metabolic), and CMS4 (mesenchymal), and each consensus molecular subtype has its own distinctive molecular, biological, and clinical features. However, the massive high-throughput gene expression profiles have brought major challenges for researchers in the design of computational models to identify cancer subtypes at different levels (Gao *et al.* 2019); For instance, Guinney *et al.* (2015) proposed a machine learning model called CMS classifier based on random forest and single-sample predictor to predict CMSs. Cascianelli *et al.* (2020) discussed the performance of regularized multiclass logistic regression (mLR) to differentiate the molecular subtypes of breast cancer. Wang *et al.* (2020) improved the random forest method for lung cancer classification. However, these computational methods using machine learning to address gene expression profiles often suffer from high dimensionality and computational scalability.

Deep learning models are made up of multiple neuron layers with non-linear activation and have been successfully applied in various fields including image processing and text classification. A few deep neural network (DNN) models have been developed to identify cancer subtypes from gene expression data; for instance, Gao *et al.* (2019) proposed DeepCC to adopt deep learning in predicting CMSs and demonstrated its competitive performance over traditional machine learning. Sirinukunwattana *et al.* (2021) proposed an image-based deep learning model for consensus molecular subtype classification of colorectal cancer. Chen *et al.* (2020) proposed to combine supervised deep learning with the unsupervised *k-means* method to predict breast cancer subtypes. Liu *et al.* (2021) developed a pipeline to classify triple-negative breast cancer by combining deep embedding learning for feature extraction with genetic algorithm for identification. The inception network is popularly adopted for identifying different cancer as it has promising performance in processing images (Banerjee *et al.* 2017, Alom *et al.* 2019). Lee *et al.* (2020) proposed a graph convolutional network based on the KEGG pathway to identify cancer subtypes. Unfortunately, the performance of most deep learning models is still under-explored and there is significant room for improvement of learning abilities. Meanwhile, most deep learning models lack of biological interpretability.

To address these challenges, we developed a fully automated deep neural architecture search model (DNAS) for diagnosing CMSs in colorectal cancer data. On the one hand, DNAS has the ability to self-execute feature engineering by scanning diverse and complex colorectal cancer data for extracting critical latent features and then integrating them to facilitate faster learning, thus effectively bridging the gap of existing machine learning methods that are insensitive to high-dimensional sparse data. The experimental results show that DNAS has better dimensionality reduction performance whether compared with traditional machine learning methods or deep learning methods. Meanwhile, compared to the deep model architectures explicitly designed for the corresponding tasks, DNAS equipped with generalization strategies allows automatic search for the best neural network architecture on a given issue, which helps non-trained biological researchers to select the optimal model hyperparameters without much effort. For T-SNE on lung cancer subtype, the results demonstrate that the latent embedding representation of DNAS is biologically interpretable, while other deep learning approaches might not. We tested DNAS on eight real datasets from CRCSC and the results demonstrated that our model provided competitive performance over other CMSs classifiers. After that, we investigated the general applicability of DNAS by identifying intrinsic subtypes of other cancer across different platforms. The experimental results on lung cancer and breast cancer indicate that DNAS has robust prediction and dimensionality reduction performance on different cancer subtypes from different cross-platform data. Subsequently, we conducted several experiments on an external cohort to validate DNAS performance and robustness. In addition, an biology analysis on the external cohort was conducted to reveal insights into colorectal cancer.

## 2 Materials and methods

### 2.1 Data collection

In this study, we collected eight independent colorectal cancer datasets with gene expression data based on Affymetrix HG133plus2 platform: GSE13067 (*n *=* *67), GSE13294 (*n *=* *140), GSE14333 (*n *=* *135), GSE17536 (*n *=* *38), GSE20916 (*n *=* *71), GSE2109 (*n *=* *266), GSE37892 (*n *=* *118), and GSE39582 (*n *=* *519). All the datasets can be downloaded from the official repository of the international CRC subtyping consortium on Synapse (https://www.synapse.org/#!Synapse: syn2623706/wiki/) (downloaded on 3 January 2021). The details of the eight datasets are summarized in Supplementary Table S1. To visualize those eight datasets, we applied four dimensional reduction methods to project the datasets onto 2D spaces as visualized in Supplementary Fig. S1. It can be seen that consensus molecular subtypes are hardly distinguishable by linear models. Therefore, it is urgent and necessary to develop nonlinear models to identify the consensus molecular subtypes from the colorectal cancer datasets. Meanwhile, we collected a The Cancer Genome Atlas (TCGA) CRC set (*n *=* *512) which based on RNA sequencing platform. The TCGA expression data can be downloaded from CRCSC Synapse repository (https://www.synapse.org/\#!Synapse: syn2623706/wiki/). The CMSs clinical labels with all CRC samples can be downloaded from CRCSC Synapse repository (https://www.synapse.org/\#!Synapse: syn2623706/wiki/).

We conducted two experiments to validate the performance of DNAS: the lung cancer study and the breast cancer study. In the lung cancer study, two lung cancer subtypes were collected from TCGA (https://portal.gdc.cancer.gov/), which consists of 415 adenocarcinoma (LUAD) and 417 squamous cell carcinoma (LUSC) samples. The visualization of lung cancer dataset is summarized in Supplementary Fig. S1, which demonstrate the two intrinsic subtypes of lung cancer is hardly diagnosed. The breast cancer data can be downloaded from the METABRIC project (Curtis *et al.* 2012), which consists of 144 normal breast tissue samples and 1989 primary breast tumor samples. The PAM50 subtypes were obtained as the class label of breast cancer. The data distribution in Supplementary Fig. S1 also demonstrate the breast cancer subtypes is very complexity and hardly distinguished.

### 2.2 Adaptive deep learning model (ADLER)

ADLER is a feed-forward DNN assembled by multilayer perceptron with adaptive loss. ADLER uses the sequential alternating representation layers with nonlinear activation functions to learn the potential features, followed by representation layers with *SoftMax* activation functions as the outputs of ADLER to compute the probability of each CMSs. Mathematically, it is defined as follows:


(1) $$f^{l}(x)=\alpha_{l}(W_{l}f^{l-1}(x)-\theta_{l}),$$

while *x* is the input of our model, $f^{l-1}(x)$ is the *l—*1-th layer’s output, *θ_l_* is the *l*-th layer’s threshold, and *α_l_* is the *l*-th layer’s activate function.

To reduce the influence of overfitting, the dropout layer and batch normalization layer were added into our model to identify CMSs. The dropout layer intended to drop some of the neuronscan, and it is described as follows:


(2) R=Bernoulli(p),

(3) $$f^{\prime }(x)=R*f(x),$$

where the Bernoulli function is used to randomly generate a binary mask vector with Bernoulli trial probability *p*, *f*(*x*) and $f^{\prime }(x)$ are the input and output of the dropout layer, respectively.

To tolerate a wide range of learning rate and relieve the impact of initialization, Batch Normalization (BN) is employed to assemble the ADLER, which is described as follows:


(4) $$\begin{array}{c} BN_{i}=\gamma \frac{x_{i}-\frac{1}{m}\sum_{i=1}^{m}x_{i}}{\sqrt{\frac{1}{m}\sum_{i=1}^{m}(x_{i}-\frac{1}{m}\sum_{i=1}^{m}x_{i}{)}^{2}+\varepsilon }}+\beta \end{array},$$

where $x_{i}\in \{x_{i}^{1},x_{i}^{2},.....,x_{i}^{m}\}$ is the input with *m* dimension, *m* is the batch size, *γ* and *β* are the trainable parameters of *BN*.

Furthermore, we integrated adaptive loss functions (*L*_1_ and *L*_2_ regularization) into the ADLER model. *L*_1_ and *L*_2_ regularizations enhance the generality of ADLER by adding a penalty term to the empirical loss. Because *L*_2_ has a closed form solution, which is a weight squared, it is generally better than *L*_1_ in terms of accuracy and much easier to optimize. On the other hand, *L*_1_ can handle sparse feature spaces, which facilitates feature selection and leads to reduction in data dimensionality (Zou and Hastie 2005). Therefore, the objective function can be defined as:


(5) $$\mathrm{loss}=\sum_{i=0}^{n}\sum_{t=0}^{3}y_{i,t}ln(y_{i,t}^{\prime })+\gamma_{1}||w|{|}_{1}+\gamma_{2}||w|{|}_{2},$$

while *n* is the number of samples, $y_{i,t}^{\prime }$ is the output of the *i*-th sample at the *t*-th neuron of the last layer from the neural network (*t* falls into 0 to 3 because there are four consensus molecular subtypes), *w* is the weight matrix of last output layer, and $\gamma_{1}||w|{|}_{1}+\gamma_{2}||w|{|}_{2}$ is the *L*_1_ and *L*_2_ regularization, respectively.

### 2.3 Nature-inspired deep neural architecture search model (DNAS)

In this section, we proposed a nature-inspired deep neural architecture search model for consensus molecular subtypes from colorectal cancer data, DNAS. DNAS automatically seeks the optimal neural architecture for the ADLER model, and consists of five important components: population initialization, ant colony optimization (ACO) (Dorigo and Gambardella 1997) global search, ACO local search, the dynamic weighting hyperparameter model, and the objective function.

#### 2.3.1 Population initialization

(1) *Population*: As mentioned in Section 2.1, the ADLER model has many hyperparameters including the number of epochs (*e*), the optimizer (*τ*), the initial learning rate for the optimizer (*α*), and the structure of hidden layers ($H=\{h_{1},h_{2},\ldots ,h_{k}\}$), for which *k* is the number of hidden layers. Specifically, the basic parameters for each hidden layer, $h_{i}\in H$ including the size of the latent space ($\bar{\vartheta }=\{\vartheta_{1},\vartheta_{2},\ldots ,\vartheta_{k}$) and the activate function ($\bar{\delta }=\{\delta_{1},\delta_{2},\ldots ,\delta_{k}\}$). However, to stabilize the training process and increase the generalization of the deep learning classifier, dropout layer ($\bar{\rho }=\{\rho_{1},\rho_{2},\ldots ,\rho_{k}\}$), BN layer ($\bar{\omega }=\{\omega_{1},\omega_{2},\ldots ,\omega_{k}\}$), *L*_1_ ($\bar{\gamma_{1}}=\{\gamma_{1_{1}},\gamma_{1_{2}},\ldots ,\gamma_{1_{k}}\}$), and *L*_2_ ($\bar{\gamma_{2}}=\{\gamma_{2_{1}},\gamma_{2_{2}},\ldots ,\gamma_{2_{k}}\}$) regular penalty terms are considered as candidate parameters for *h_i_*. To guarantee each individual consists of all parameters, we designed a hybrid encoding process to represent the hyperparameters as $p_{i}=[e,\tau ,\alpha ,\bar{\vartheta },\bar{\delta },,\bar{\rho },\bar{\omega },\bar{\gamma_{1}},\bar{\gamma_{2}}]$.

After that, DNAS creates a directed graph network under the fixed hyperparameter pool. Then, a population with *n* individuals $P=\{p_{1},p_{2},\ldots ,p_{n}\}$ is generated to represent the parameters of different ADLER frameworks according to the hybrid encoding rule. For each individual $p_{i}\in P$, ants select the hyperparameters to be visited through a stochastic mechanism. The probability of the individual *p_i_* can be defined as follows:


(6) $$p_{ij}^{s}=\left\{\begin{array}{cc} \frac{\varrho_{ij}^{\bar{\alpha }}\cdot \eta_{ij}^{\bar{\beta }}}{\sum_{c_{il}\in N(p\prime )}\varrho_{il}^{\bar{\alpha }}\cdot \eta_{il}^{\bar{\beta }}} & \text{if}c_{il}\in N(p\prime )\\ 0 & \text{otherwise} \end{array}\right.,$$

where N(p′) is the set of feasible hyperparameters consisting of edges (*i*, *l*) representing the relationship between the hyperparameter *i* and the hyperparameter *l* not yet visited by the individual *p_i_*, $\bar{\alpha }$ and $\bar{\beta }$ control the relative impact between the pheromone and versus the heuristic information *η_ij_*.

*(2) Encoding*: The encoding process operates on the trainable DNAS whose architectures are generated by the ACO algorithm. To encode the DNAS architecture appropriately for subsequent processing, the non-numeric parameters are considered as a one-hot vector. Based on Section 2.1, the encoding process for each individual p∈P is summarized as Algorithm 1.

Algorithm 1: Encoding Operator

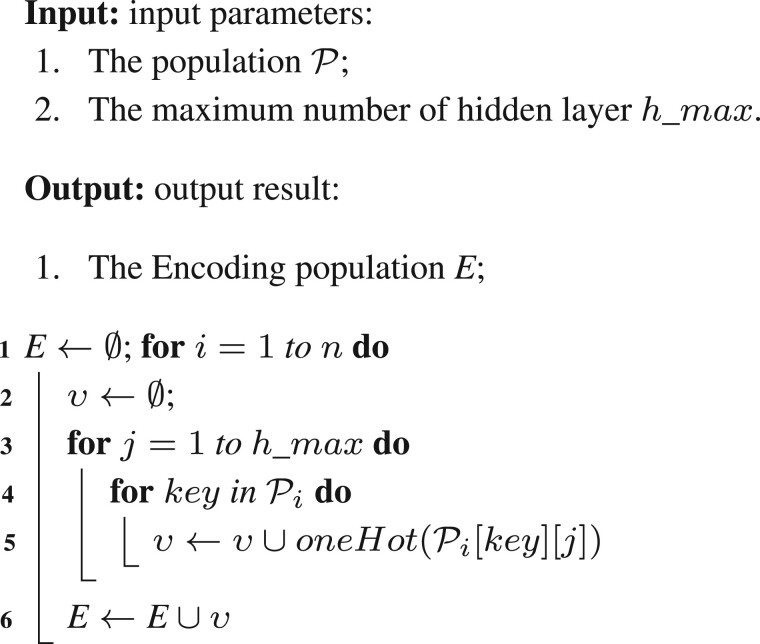

#### 2.3.2 ACO global search

In our study, only the best ant is allowed to deposit pheromone. The ACO global search is described as follows:


(7) $$\begin{array}{c} \xi \left(r,s\right)\leftarrow \left(1-\varphi \right)\xi \left(r,s\right)+\varphi *\Delta \xi \left(r,s\right)\\ \Delta \xi (r,s)=\{\begin{array}{cc} \eta (r,s)*f(p) & \text{if}\;(r,s)\in p\\ 0 & \text{otherwise} \end{array} \end{array}.$$

0<φ≤1 is the pheromone decay parameter, *p* is the best individual allowed to deposit pheromone, 0<f(p)≤1 is the objective function for *p*. Equation (7) is executed only after all ants have finished their tours. This global updating rule guarantees that only the edges existing in the globally best path will be strengthened.

#### 2.3.3 ACO local search

The ACO local search is proposed at the end of each construction step. The algorithm will yield different ADLER frameworks by reducing the concentration of pheromones on the selected hyperparameters. The ACO local search is defined as:


(8) $$\xi (r,s)\leftarrow \left\{\begin{array}{cc} \left(1-\psi )*\xi (r,s)+\psi *\Delta \xi (r,s\right) & if\;(r,s)\in p\\ \xi (r,s) & \mathrm{otherwise} \end{array}\right.,$$

where 0<ψ≤1 is the pheromone decay coefficient.

#### 2.3.4 Dynamic weighted hyperparameter model

In this section, we describe employing XGBoost to quantify the impact of each hyperparameter on ADLER to guide ACO iteration. The dynamic weighted hyperparameter model is summarized in Algorithm 2.

Algorithm 2: Dynamic weighted hyperparameter model

 **Input**: input parameters:

 1. the encoded ADLER architecture for population $E(P)=\{E(p_{1}),\ldots ,E(p_{n})\}$;

 2. the objective function value for population $f(P)=\{f(p_{1}),\ldots ,f(p_{n})\}$;

 **Output**: output result:

  1.  The hyperparameter weight ϕ;

  2.  The XGBoost regression score *ν*;

**1** T←XGBoost() ;

2 T←T(E(P),f(P));

3 $\phi \leftarrow T_{\phi }$;

4 ν←metrics(T(E(P),f(P))).

1. *XGBoost:* We employ XGBoost as the dynamic weighting hyperparameter model for the following reasons; as DNAS encodes each $p_{1}\in P$ based on the principle of one-hot, E(P) consists of many zeros. XGBoost provides a boosting algorithm for handling such sparse data (E(P)) to obtain a more robust performance. Meanwhile, XGBoost provides a regularization term in its loss function to avoid overfitting.
2. *Dynamic weighting hyperparameter*: To maintain the real-time validity of surrogates, XGBoost is re-trained at each iteration. Then, DNAS determines the weight of each pair of hyperparameters for ADLER through comprehensive consideration of the feature importance, as formulated below:
  (9) η(r,s)=ν*ϕ,

where *ν* is the regression performance of XGBoost, ϕ is the feature importance score computed by trained XGBoost for each hyperparameter. After that, η(r,s) is adopted to update the pheromone for the ACO Search stages.

#### 2.3.5 Objective function

Generally, for cancer subtype diagnosis models, accuracy is widely used to quantify the performance of each individual in the population, which can be defined as follows:


(10) $$\mathrm{Accuracy}=\frac{1}{n}\sum_{i=1}^{n}I(y_{i}=y_{i}^{*}),$$

where *I*(*x*) is the indicator function, *y* is the truth label and $y^{*}$ is the corresponding diagnostic labels. Therefore, the objective function in our study is to maximize Equation (10).

#### 2.3.6 Termination criteria

To comprehensively obtain a promising performance of DNAS, we develop two termination conditions. Firstly, a single ADLER training process will be terminated if the performance on the validation set does not improve over 50 epochs or the maximum of epochs. Secondly, the termination condition is to reach 10 iterations in the evolution process.

#### 2.3.7 Performance evaluation

Three evaluation metrics are used in this study and are defined as follows:


(11) Sensitivity=TP/(TP+FN),

(12) Specificity=TN/(TN+FP),

(13) $$\mathrm{Accuracy}=\frac{1}{n}\sum_{i=1}^{n}I(y_{i}=y_{i}^{*}),$$

where *I*(*x*) is the indicator function, *y* is the truth label and $y^{*}$ is the corresponding diagnostic label, and *n* is the number of samples, TP is the true positive, TN the true negative, FP the false positive, and FN the false negative.

#### 2.3.8 Time complexity analysis

In this work, the time complexity mainly depends on the complexity of ADLER and the evolutionary steps. For ADLER, the time complexity is $O(n^{2}+O(n\times m\times k))$ using the stochastic gradient descent optimizer, where *n* represents the number of samples, *m* is the feature size, *k* is the number of iterations. For ACO, the time complexity is O(N×D), where *N* is the population size, *D* is the dimension of the hyperparameter for a single ADLER. Therefore, the overall time complexity of DNAS is $O(N\times (n^{2}+n\times m\times k))$ in the worst situation.

### 2.4 Parameter settings

In DNAS, we chose different hyperparameters from different pools to assemble a single ADLER architecture. Especially, the hyperparameters lambda1 and lambda2 are tuned on validation sets by 10-fold cross-validation, and after they are tuned, the model was applied on test datasets to generate test accuracy for comparison. The details are described in the Supplementary File.

## 3 Results

### 3.1 Methodology overview of DNAS

**Figure 1.**
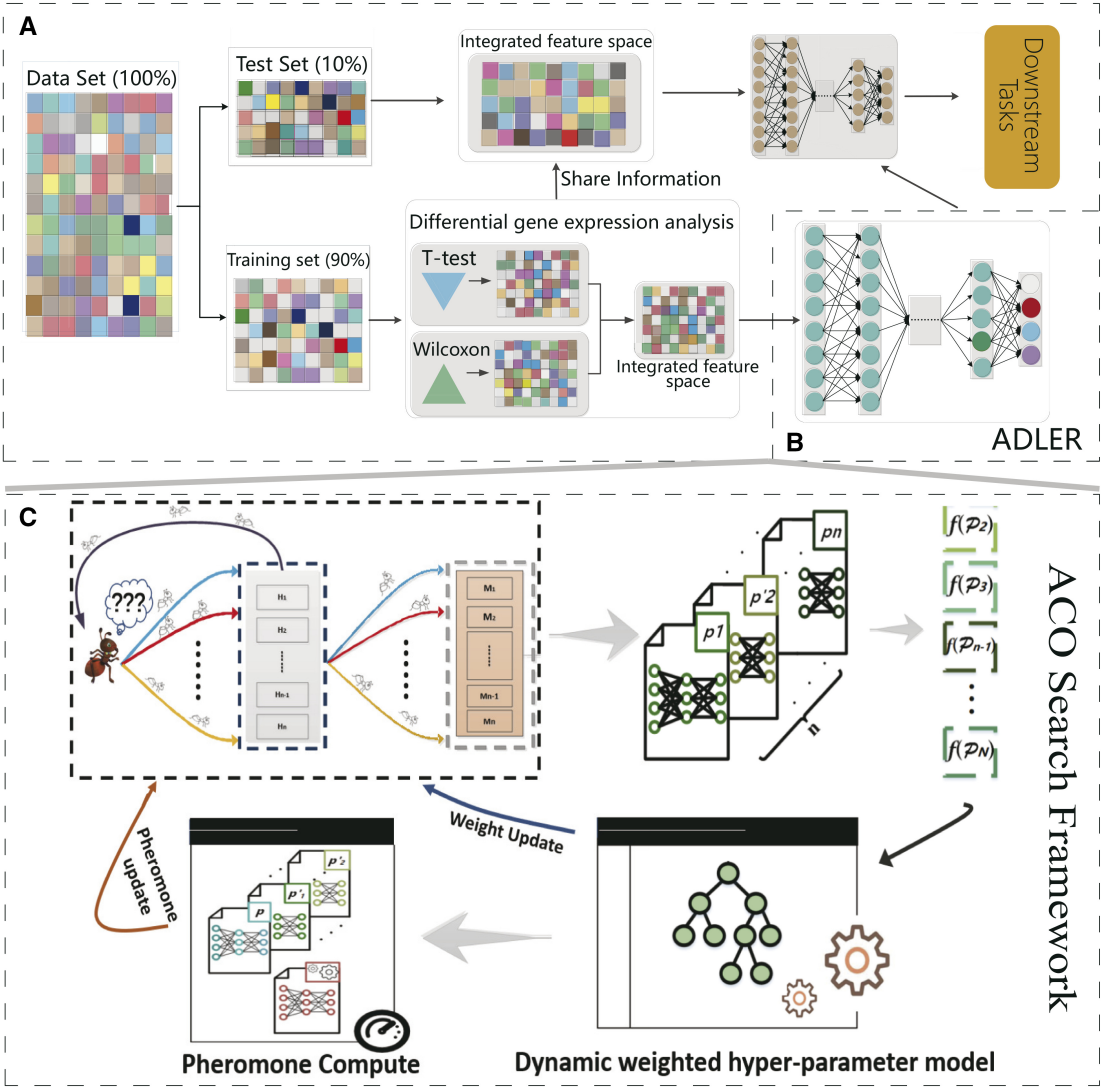
Overview of the proposed method, DNAS, for diagnosing CMSs in colorectal cancer. (A) The framework of DNAS, to maintain the biological significance, we integrate the T-test and Wilcoxon together to analyze differentially expressed genes (DEGs). (B) The learning model (ADLER) with integrated feature space are employed for cancer subtype identification. (C) The fully automated deep neural network searching framework based on ant colony optimization (ACO) algorithm. It includes population initialization, ACO global search, ACO local search, dynamic weighting hyper-parameter model, and pheromone update. This ACO global search guarantees that only the edges existing in the globally best path will be strengthened. The ACO local search will yield different ADLER frameworks by reducing the concentration of pheromones on the selected hyperparameters. Using dynamic weighted hyper-parameter model to quantify the impact of each hyperparameter on ADLER to guide ACO iteration. The optimal results are obtained by global updating with pheromone and weight, where pheromone compute based on the distance traveled by each ant on the path and fitness.

In this section, we propose an automated nature-inspired DNN architecture search model for cancer subtype identification called DNAS which is shown in Fig. 1. Considering a gene expression data matrix, $X=\{X_{1},X_{2},\ldots ,X_{i},\ldots ,X_{n}\},i=1,2,....,n$ and then consensus molecular subtypes $y_{i}\in Y$, our model is trained on X to diagnose Y through fully-connected layers and the adaptive loss to obtain the feature representation of X in Phase A.

Then, to alleviate the impact of high-throughput gene expression profiles, discriminative gene expression analysis is adopted to select important genes from colorectal cancer data in phase A, which are then fed into Phase B.

After that, we propose DNAS, a pipeline based on ACO for automatically discovering the best architecture of ADLER to identify consensus molecular subtypes from colorectal cancer gene expression profiles and then apply XGBoost to analyze the feature importance and then assign different weights to each hyperparameter. In Phase C, a population $P=\{p_{1},p_{2},\ldots ,p_{n}\}$ is proposed to represent the hyperparameters of the ADLER framework including the number of epochs (*e*), the optimizer (*τ*), the initial learning rate for the optimizer (*α*), and the structure of the hidden layers ($H=\{h_{1},h_{2},\ldots ,h_{k}\}$). Each hidden layer $h_{i}\in H$ includes the size of the latent space ($\bar{\vartheta }$) and the activate function ($\bar{\delta }$). Moreover, to prevent overfitting of the algorithm and thus improve the generalization of deep learning, the dropout layer ($\bar{\rho }$), Batch Normalization (BN) layer ($\bar{\omega }$), *L*_1_ ($\bar{\gamma_{1}}$) and *L*_2_ ($\bar{\gamma_{2}}$) regular penalty terms are considered as candidate parameters for *h_i_*.

### 3.2 DNAS is the most accurate among identification methods on colorectal cancer

To demonstrate the performance of DNAS, six machine learning methods including Random Forest (RF) (Breiman 2001), XGBoost (Chen and Guestrin 2016), Gradient Boosted Decision Trees (GBDT) (Friedman 2002), Elastic Linear Model (ELM) (Zou and Hastie 2005), Support Vector Machine (SVM) (Hsu and Lin 2002), and CMSclassifier (Guinney *et al.* 2015) were compared to DNAS. In addition, we also compared three deep learning models including DeepForest (Zhou and Feng 2019), DeepCC (Gao *et al.* 2019), and Inception_Res (Szegedy *et al.* 2017). Meanwhile, we added a deep learning model with two hidden layers (500, 30) with *Tanh* activation without the evolutionary optimization, named ADLER1, to demonstrate the effectiveness of our evolutionary framework.

**Figure 2.**
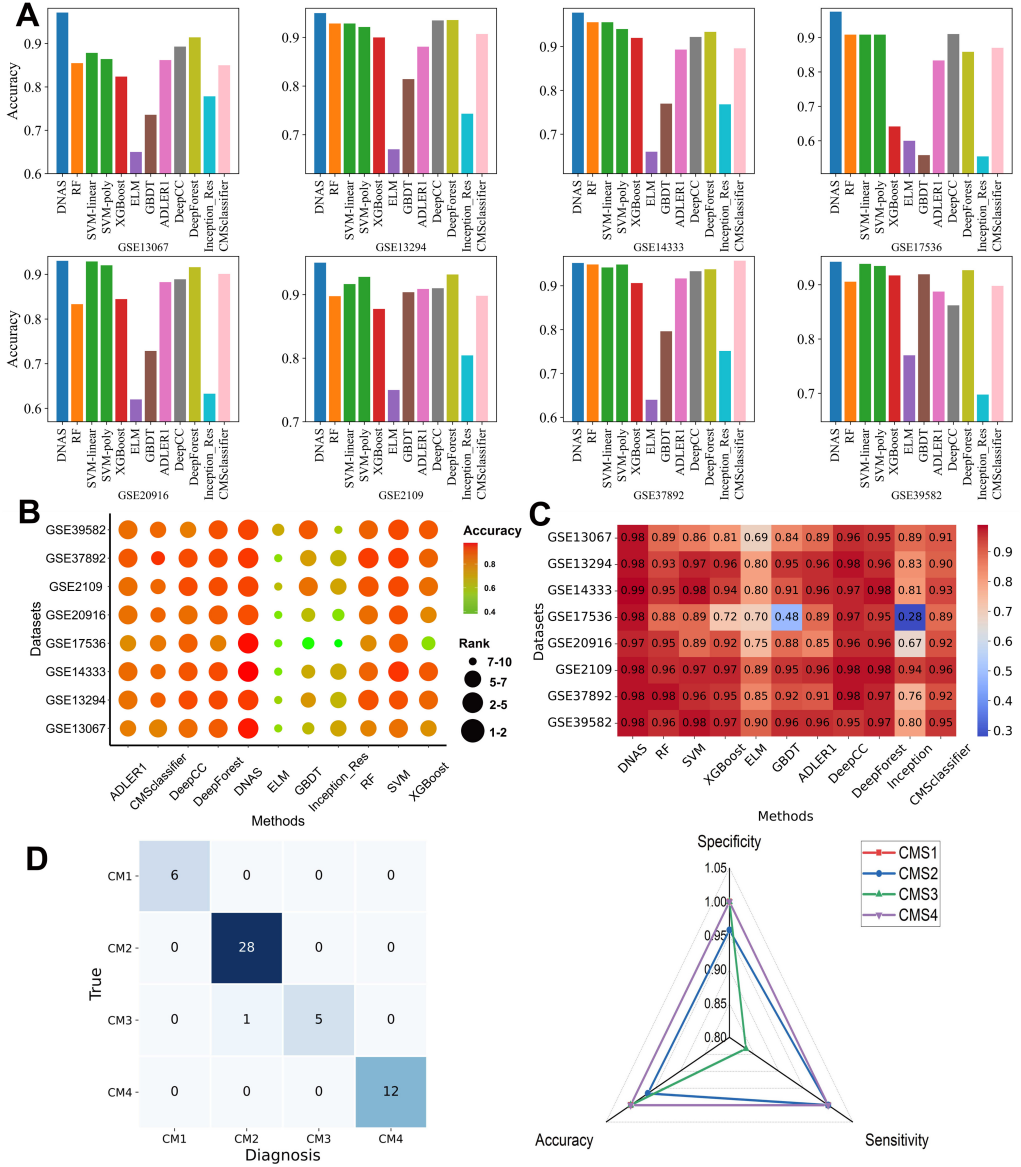
Performance comparisons on eight colorectal cancer datasets and a TCGA dataset. (A) Accuracy (calculated by the average accuracy per class). (B) Dot plot of average accuracy. Each point on the *X*-axis represents a method, *Y*-axis represents a dataset. (C) Specificity (calculated by the average specificity per class). (D) The confusion matrix and performance of DNAS on TCGA dataset.

In the first, we compared our proposed model with machine learning models. The experimental results are summarized in Fig. 2; it can be observed that our proposed DNAS achieves the best performance. Regarding accuracy, DNAS was the only one to achieve 90% on all datasets and its average accuracy was 4%–29% higher than other six methods in Fig. 2A and B. For specificity, DNAS also outperformed the other competitive methods. From Fig. 2C, the average specificity values of DNAS are 2%–18% higher than other six methods. For the Supplementary Fig. S2, DNAS was the only model to achieve 95% on the colorectal cancer datasets. Therefore, DNAS provided a more competitive performance than the six machine learning methods on eight colorectal cancer datasets. Further, we compared the performance of our algorithm to four different deep learning models under 10-fold cross-validation. The experimental results are summarized in Fig. 2. We observed that DNAS achieves the best performance while Inception_Res yield the worst. For the accuracy, DNAS was the only model with above 90% performance on all datasets. In particular, the performance of DNAS was superior to ADLER1 on all datasets, indicating that the ACO algorithm can optimize the ADLER architectures on all datasets. For the specificity and sensitivity, DNAS also outperformed other competitive deep learning models. In summary, we conclude that DNAS provides better performance than four other deep learning models and could unleash its power to deal with the colorectal cancer.

After that, to demonstrate the robustness of DNAS, we also applied DNAS to annotate cancer subtypes on an independent colorectal cancer dataset from the Cancer Genome Atlas (TCGA) (Guinney *et al.* 2015). The TCGA expression data can be downloaded from (https://www.synapse.org//#!Synapse:syn2623706/wiki/). The TCGA dataset has 512 patients with explicit CMSs labels and each patient sample has 20293 features. At this point, we randomly left out 10% of the samples to construct a test set. Then, we trained DNAS on the remaining 90% samples by 10-fold cross-validation. The results are summarized in Fig. 2D. We observe that only one sample from CMS3 is incorrectly diagnosed as CMS2. The performance of DNAS was not degraded by the transformation of the data protocol, demonstrating the robustness of DNAS on different cross-platform tasks.

### 3.3 Evaluation of parameter selection and feature importance analysis

To determine the best number of decision trees in our DNAS framework, we tested ten different numbers of decision trees, 200, 400, 600, 800, 1000, 1200, 1400, 1600, 1800, 2000, named DT1, DT2, DT3, DT4, DT5, DT6, DT7, DT8, DT9, DT10, respectively. The left figure Supplementary Fig. S3 summarizes the average accuracy of different numbers of decision trees on all 8 colorectal datasets. From the experimental results, we conclude that the average accuracy of DT5 is higher than the other decision tree numbers. Therefore, we chose the number of decision trees to be 1000 in DNAS.

To assess the robustness of DNAS, we conducted a series of experiments on 8 colorectal cancer datasets under the same termination condition. We reran DNAS on the colorectal cancer datasets to measure the performance of different population sizes {5,10,15,20}, namely PA1, PA2, PA3, and PA4, in 100 fitness function evaluations, the experimental results are summarized in the right figure of the Supplementary Fig. S3 where we observe that PA2 provides the best average accuracy.

After that, we investigated the effects of six gene selection methods on the model. To maintain the biological significance, we integrate the *T-test* and *Wilcoxon* together to analyze differentially expressed genes (DEGs). Indeed, we combine the subtypes into four groups: CMS1 versus others, CMS2 versus others, CMS3 versus others, and CMS4 versus others. Then, DEGs are identified based on $\log{}_{2}$ fold-change and *Q* values under *T-test* and *Wilcoxon*, respectively. Specifically, the genes with $|\log{}_{2}$ fold-change|>1 and Q<0.05 are retained and identified as DEGs. Finally, the screened DEGs are used as input for the ADLER model.

**Figure 3.**
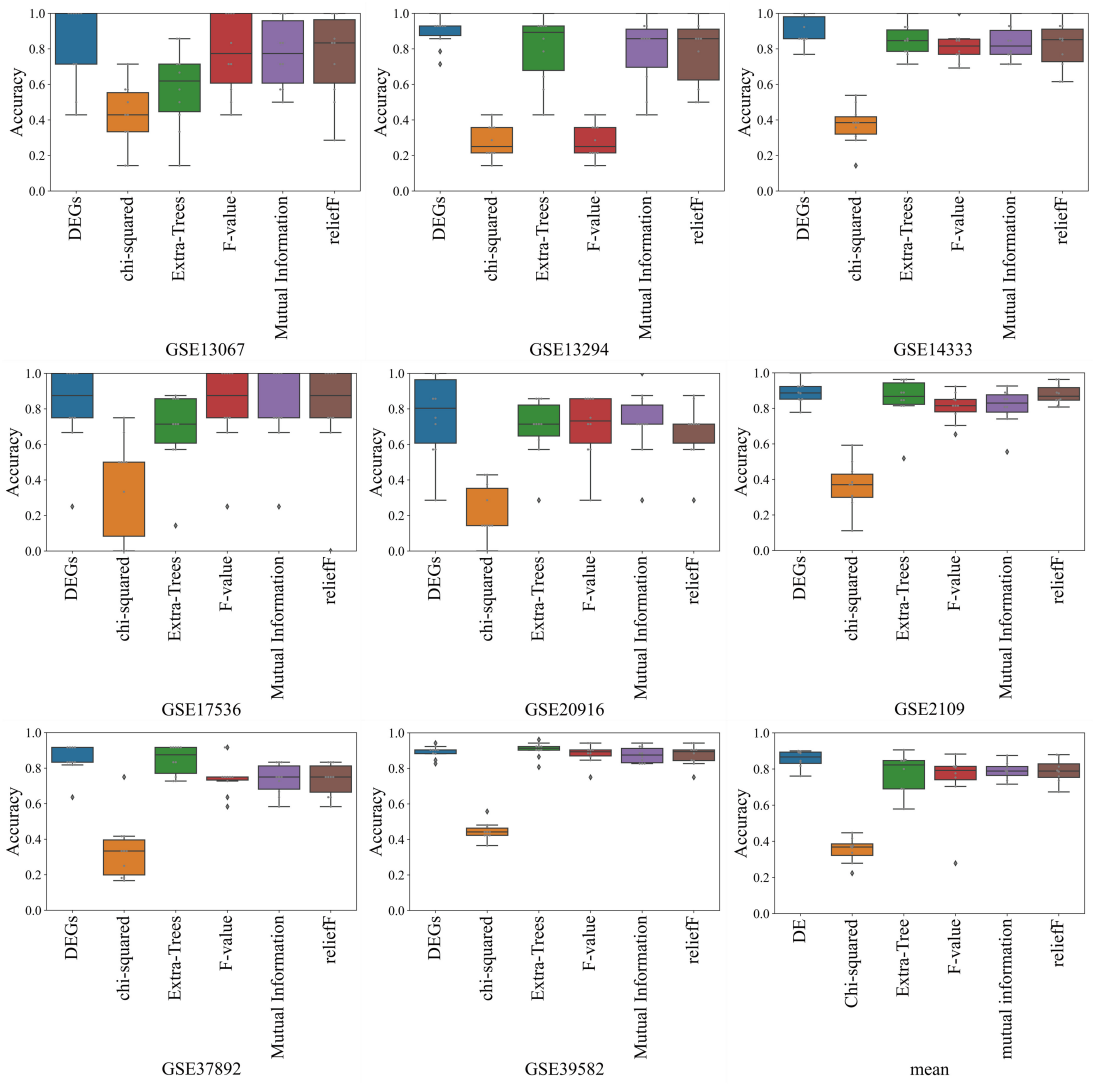
The performance comparisons of ADLER1 with six distinctive feature selection methods including DEGs, Extra-Trees, chi-squared, F-value, Mutual Information, and ReliefF.

To demonstrate the effectiveness of the differential gene expression analysis model in our study, other five feature selection models including Extra-Trees, chi-squared, F-value, Mutual Information, and ReliefF were employed as comparison. The experimental results are depicted in Fig. 3, which reveal that the differential gene analysis model provides better performance than the other models, enhancing the performance for the whole identification framework. Therefore, we can conclude that differential gene expression analysis is an inevitable step toward a more precise diagnosis of cancer subtypes.

### 3.4 DNAS can detect lung cancer subtypes from different cross-platform data

In 2020, about 2.2 million new lung cancer patients have been diagnosed and there were ∼1 800 000 deaths around the world. To elaborate the general applicability of DNAS, we investigated the performance of DNAS on lung cancer detection. Indeed, we collected two most prevalent subtypes of lung cancer from TCGA including LUAD and LUSC, whose distinction requires visual inspection by an experienced pathologist (Coudray *et al.* 2018).

**Figure 4.**
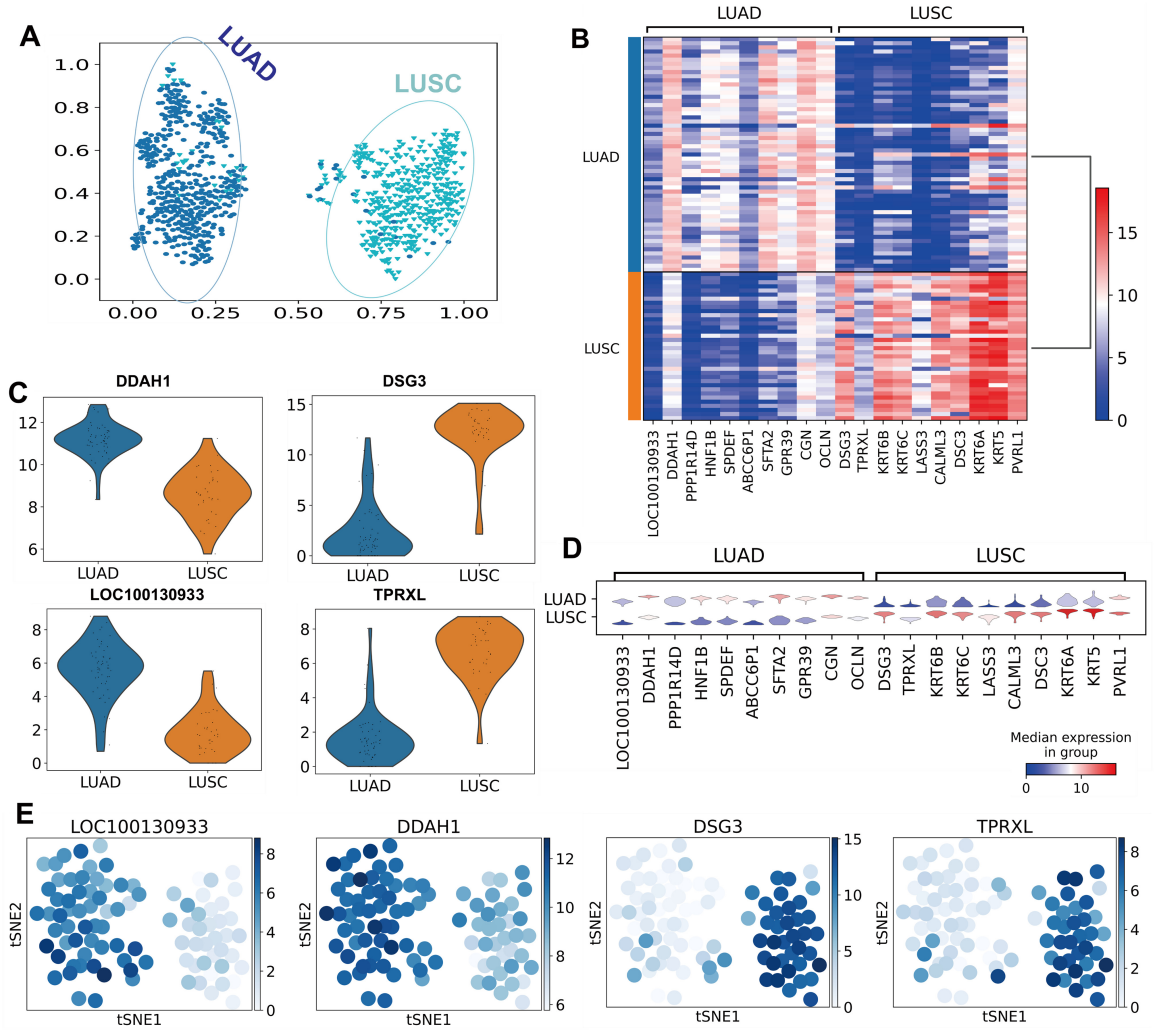
The analysis of DNAS on lung cancer. (A) T-SNE visualization of lung cancer data after gene selection. (B) The heat map of top 10 marker genes expression values in each lung cancer subtype identified by DNAS. (C and D) The count expression visualization of top 10 marker genes in LUAD and LUSC identified. (E) Visualization of samples coming from top 2 marker genes in LUAD and LUSC identified.

We collect a lung cancer dataset with 925 samples and 20 486 genes. Firstly, we trained DNAS on a training set which is composed of randomly selected 832 samples. Then, the remaining 93 samples were used as the test data. Under the T-SNE methods, LUAD and LUSC of lung cancer after the DEGs scanning algorithm were identified as shown in Fig. 4A. From the results, we can observe that our DEGs model can clearly distinguish different lung cancers. In the Supplementary Fig. S4 represents the area under the receiver operating characteristics (AUROC) of DNAS on the test set. DNAS provided 98% area under the curve (AUC) on both LUAD and LUSC, which represents our model can learn the potential feature from lung cancer data. Then, we investigated the top 10 genes marked by Wilcoxon rank-sum test further. Figure 4B–E summarized the maker gene analysis on the DNAS-identified lung cancer subtypes, respectively. In particularly, *LOC100130933* and *DDAH1* were exhibited relatively high expression in LUAD (Joehanes *et al.* 2016, Huang *et al.* 2020), while *DSG3* and *TPRXL* were expressed at high levels in LUSC (Savci-Heijink *et al.* 2009, Sui *et al.* 2017). Therefore, *LOC100130933* and *DDAH1* may stimulate the growth of adenocarcinoma in lung cancer, and *DSG3* and *TPRXL* can contribute to mark squamous cell carcinoma in lung cancer.

### 3.5 DNAS to improve end-to-end breast cancer screening

In this study, we employ DANS to diagnose breast cancer to further elaborate the general applicability of our method. We adopted the PAM50 subtypes as the class labels, which have five intrinsic molecular subtypes including Basal-like, Her2+, Luminal A, Luminal B, and Normal-like.

**Figure 5.**
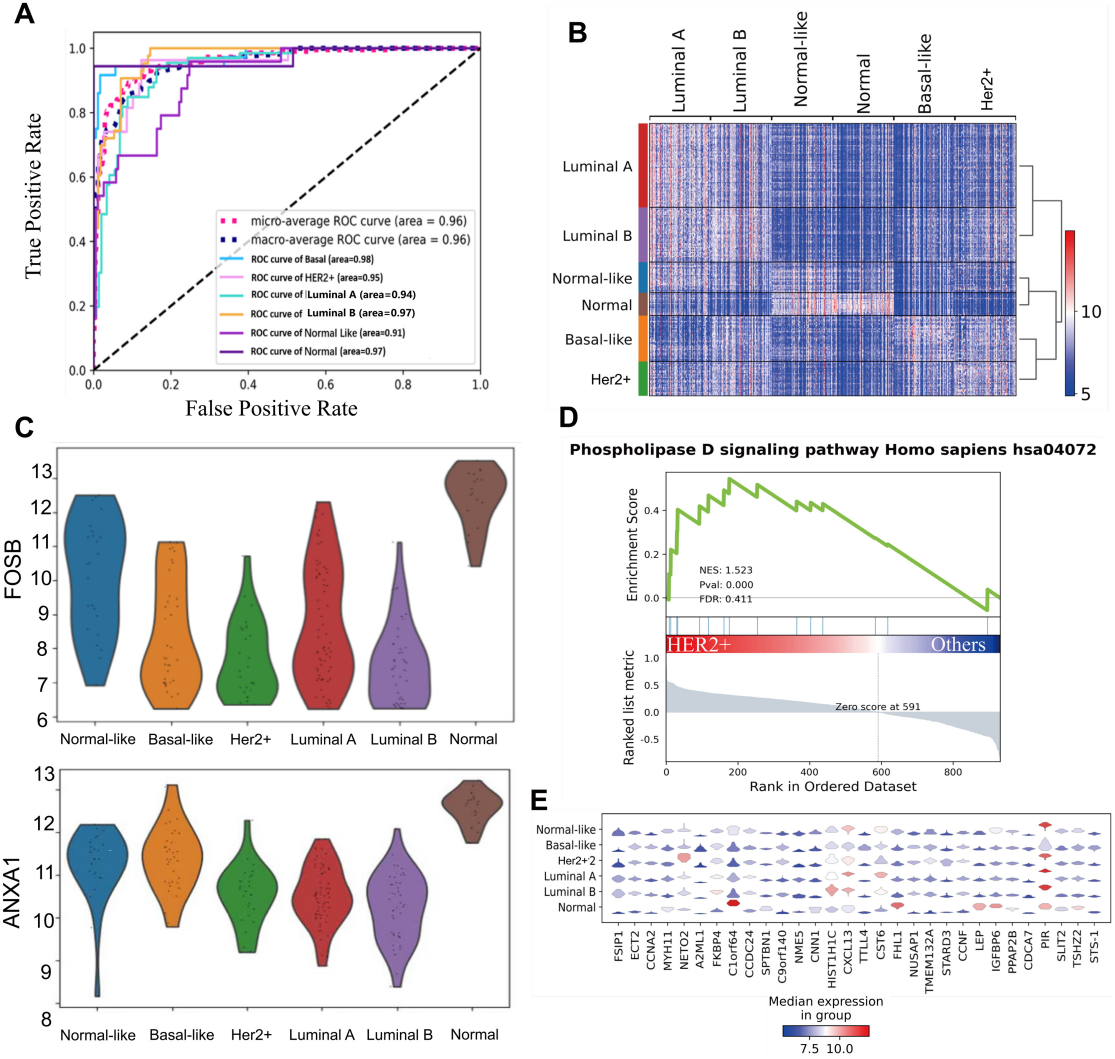
The analysis of DNAS on breast cancer. (A) The area under the receiver operating characteristics of DNAS on the test set. (B) The gene expression heat map of top 100 genes in the test set. (C) The gene count expression of FOSB and ANXA1 across six breast cancer types identified. (D) GSEA plot illustrating a representative pathway dysregulated in HER2+ identified. (E) The expression of top 5 genes in the test set.

The breast cancer dataset with six molecular subtypes includes 144 normal breast tissue samples and 1989 primary breast tumor samples from METABRIC project (Curtis *et al.* 2012). We randomly partitioned the data into training and test sets in 9:1. After the discriminative gene expression analysis, only 932 genes were scanned as inputs into DNAS. The results of the test set are summarized in Fig. 5A and Supplementary Fig. S5. As expected, DNAS achieved accuracy higher than 80% on the test set. For the AUROC shown in Fig. 5A, DNAS achieved 90% AUC for the six breast cancer subtypes. In particularly, DNAS identified 17 outs of 18 normal sample from tumor samples, which achieves 0.97 AUC. To directly assess the separation between the normal samples and tumors, we have used the Wilcoxon rank-sum test for visualizing the expression of top two marker genes, which are summarized in Fig. 5C. The normal samples expressed relatively high levels of *FOSB* and *ANXA1* (Milde-Langosch 2005, Moraes *et al.* 2018), implying that the expression of these two genes decreases after the occurrence of breast cancer. Therefore, *FOSB* and *ANXA1* can be considered as the important marker to distinguish normal sample from breast cancer. After that, the distribution of top 100 marker gene were visualized in Fig. 5B, and the expression of top five marker genes were depicted in Fig. 5E. Meanwhile, we have targeted the dysregulated pathways in each breast cancer subtype by gene set enrichment analysis (GESA), summarized in Fig. 5F and Supplementary Fig. S7. The most statistically significant pathways identified by each subtype showed that Basal was characterized by MicroRNAs in cancer (Milioli *et al.* 2017); HER2+ was characterized by the phospholipase D signaling pathway (Cho and Han 2017); Luminal A was characterized by HTLV-I infection (Hirata *et al.* 2019); Luminal B by transcriptional dysregulation in cancer (Betts *et al.* 2013); Normal-like was characterized by the phospholipase D signaling pathway (Cho and Han 2017); and normal was characterized by focal adhesions (Luo and Guan 2010), which has been demonstrated that those five significant pathways are extremely important in the initiation, progression and metastasis of breast cancer (Rodrigues *et al.* 2007, Feng *et al.* 2018).

### 3.6 External cohort studies

To further evaluate performance of DNAS in a fair manner, we also conducted two experiments on an external cohort analysis by completely excluding GSE14333 (135 out of 1354 samples, ∼10%) named EC1, and GSE2109 (266 out of 1354 samples, ∼20%) named EC2. Meanwhile, the technical batch effect of all the datasets were corrected as the way in Guinney’s work (Guinney *et al.* 2015), the results were shown in Supplementary Fig. S9. Additionally, we also compared our proposed model to eight other competitive models, including SVM, XGBoost, RF, ELM, GDBT, DeepForest, DeepCC, Inception_Res, as visualized in Supplementary Fig. S6A. We observe that DNAS produced the best performance in terms of accuracy, specificity, and sensitivity. On EC1, as shown in the left of the Supplementary Fig. S6A, the accuracy of DNAS outperformed other methods by 1.5%–10%. On EC2, as shown in the right of the Supplementary Fig. S6A, DNAS was the only method that gave 90% accuracy. We further conducted the comparison experiments on other datasets following the same strategy used above, i.e. training the model on a comprehensive cohort except for one of the colorectal cancer datasets, and test the model on that remaining dataset. Obviously, DNAS still maintained favorable robustness and achieved the best performance as shown in Supplementary Fig. S8. Therefore, we can conclude that our proposed DNAS is more robust than other eight methods and may provide a reference for the deployment of automatic and robust models for the diagnosis of colorectal cancer subtypes.

### 3.7 Genomic interpretability

In the previous section, we conduct several experiments that demonstrate the promising performance of DNAS. Next, we investigated the biological significance of DNAS for the external cohort in our study. Firstly, we identified the top 200 genes with the largest weight variances in the first representation layer. In principle, these genes are a “sufficient and necessary” set to represent the model’s inputs (Tran *et al.* 2021). Then, multiple enrichment analyses were conducted to elucidate the biological functions. The details were given in Supplementary file.

## 4 Conclusion

Given the central importance of cancer, consensus molecular subtype identification is desirable for patient stratification. Specifically, the most important component of consensus molecular subtyping is the ability of the underlying classifier to correctly diagnose each subtype. In this study, we proposed an automated DNN architecture search framework based on differential gene expression analysis to find consensus molecular subtypes in colorectal cancer data.

Based on our results, we observed that DNAS achieved better colorectal cancer subtype identification than eight competitive models. We also demonstrated the significance of differential gene expression analysis compared to other feature selection algorithms, as highlighted in Fig. 3. where it is obvious that differential gene expression analysis is necessary and important for cancer subtype diagnosis. In essence, DNAS brings the following advantages: (i) molecular interpretability as DNAS considers all DEGs as inputs, which is necessary to expose the integrity of the pathology. (ii) In dealing with cross-platform cancer gene expression data. (iii) Generalization ability as DNAS achieves high performance on other types of cancer.

In future work, we believe that our automated deep learning search framework approach can demonstrate its superior performance and applicability in genomics across different contexts of high-throughput molecular data.

## Supplementary Material

btad654_Supplementary_Data
