# Supplementary material for: Automated exploitation of deep learning for cancer patient stratification across multiple types
Source: Bioinformatics. 2023 Nov 2;39(11):btad654. doi: 10.1093/bioinformatics/btad654 (PMC10636288; doi:10.1093/bioinformatics/btad654)
Supplement: btad654_Supplementary_Data [file btad654_supplementary_data.pdf]

Supplementary Data:

Automated Exploitation of Deep Learning for Cancer Patient Stratification across Multiple Types

Pingping Sun [1], Shijie Fan [1], Shaochuan Li [1,2] , Yingwei Zhao [1], Chang Lu [1,3,*], Ka-chun Wong [4] and Xiangtao Li [2,*]

[1] School of Information Science and Technology, Northeast Normal University, Jilin, China.

[2] School of Artificial Intelligence, Jilin University, Jilin, China.

[3] School of Psychology, Northeast Normal University, Jilin, China.

[4] Department of Computer Science, City University of Hong Kong, Hong Kong SAR.

Supplementary Table S1: Summary of the CRC dataset

Dataset	Platform	Tissue	Total	with CMS*	Source	Number of genes
GSE13067	HG133plus2	Fresh frozen	74	67	CRCSC	54675
GSE13294	HG133plus2	Fresh frozen	155	140	CRCSC	54675
GSE37892	HG133plus2	Fresh frozen	130	118	CRCSC	54675
GSE39582	HG133plus2	Fresh frozen	566	519	CRCSC	54675
GSE2109	HG133plus2	Fresh frozen	293	266	CRCSC	54675
GSE14333	HG133plus2	Fresh frozen	157	135	CRCSC	54675
GSE17536	HG133plus2	Fresh frozen	177	38	CRCSC	54675
GSE20916	HG133plus2	Fresh frozen	90	71	CRCSC	54675

Supplementary Table S2: The parameters used by methods on each dataset

**Optimizer**	**Search space**			
DNAS	Number neurons=768	Dropout rate=0.3	L1 regulation rate: 1e-05	L2 regulation rate: 1e-02
	Batch Normalization layer=0	Activation function=Selu	Loss function= categorical_crossentropy	Optimizer=Adamax
	Learning rate=1e-05	Batch size= 256	Number layers=3	
SVM	kernel=[linear, poly]	C=1		
RF	max_depth=[3, 4, 5]	n_estimators=[50, 100, 150, 200]		
XGBoost	colsample_bytree=[0.7, 0.8, 0.9]	gamma=[0, 1, 3, 5]	learning_rate=[0.01, 0.05, 0.1]	max_depth=[3, 5, 7]
	n_estimators=[50, 100, 150, 200]	subsample=[0.7, 0.8, 0.9]		
ELM	n_hidden=[50,150,300,500]	alpha=[0.001, 0.01, 0.1]		
GBDT	max_depth=[3, 4, 5]	n_estimators=[100, 200, 300]		
DeepForest	max_depth=[5, 15, 25]	n_bins=[5, 15, 20, 25]	n_estimators=[20, 50, 100, 200]	
ADLER1	Two hidden layers=(500, 30)	activation='Tanh'		
Inception_Res	weights='imagenet'	depth=299	activation='softmax'	

Supplementary Table S3: The accuracy comparisons of L1 and L2 regularization rates for thirty-six different combinations of the eight GSE datasets with ten other methods.

Methods		GSE13067	GSE13294	GSE37892	GSE39582	GSE2109	GSE14333	GSE17536	GSE20916
DNAS									
**L1 regularization**	**L2 regularization**								
1e-5	1e-2	0.97	0.95	0.98	0.97	0.93	0.94	0.96	0.94
0	0	0.87	0.78	0.91	0.84	0.86	0.82	0.84	0.89
0	1e-1	0.83	0.85	0.95	0.87	0.93	0.91	0.88	0.85
0	1e-2	0.96	0.94	0.93	0.92	0.87	0.82	0.82	0.88
0	1e-3	0.89	0.89	0.95	0.92	0.87	0.83	0.85	0.78
0	1e-4	0.94	0.93	0.97	0.81	0.92	0.92	0.93	0.95
0	1e-5	0.93	0.95	0.85	0.89	0.93	0.94	0.82	0.88
1e-1	0	0.92	0.91	0.95	0.92	0.94	0.85	0.93	0.85
1e-1	1e-1	0.85	0.87	0.89	0.93	0.92	0.93	0.89	0.86
1e-1	1e-2	0.91	0.90	0.91	0.93	0.85	0.95	0.96	0.91
1e-1	1e-3	0.89	0.91	0.95	0.96	0.83	0.93	0.91	0.81
1e-1	1e-4	0.90	0.89	0.97	0.94	0.85	0.89	0.93	0.84
1e-1	1e-5	0.89	0.93	0.92	0.91	0.84	0.88	0.85	0.86
1e-2	0	0.92	0.85	0.81	0.95	0.89	0.86	0.82	0.91
1e-2	1e-1	0.93	0.93	0.86	0.89	0.92	0.95	0.91	0.85
1e-2	1e-2	0.95	0.94	0.87	0.98	0.91	0.82	0.85	0.84
1e-2	1e-3	0.94	0.89	0.92	0.89	0.85	0.89	0.88	0.91
1e-2	1e-4	0.92	0.82	0.89	0.83	0.94	0.85	0.95	0.92
1e-2	1e-5	0.89	0.85	0.93	0.91	0.89	0.93	0.91	0.86
1e-3	0	0.91	0.86	0.95	0.93	0.91	0.89	0.85	0.84
1e-3	1e-1	0.95	0.91	0.91	0.93	0.83	0.91	0.89	0.87
1e-3	1e-2	0.96	0.90	0.91	0.94	0.86	0.85	0.87	0.90
1e-3	1e-3	0.97	0.81	0.92	0.85	0.91	0.91	0.82	0.91
1e-3	1e-4	0.91	0.91	0.88	0.86	0.95	0.88	0.91	0.90
1e-3	1e-5	0.85	0.92	0.93	0.89	0.87	0.89	0.85	0.84
1e-4	0	0.82	0.80	0.81	0.89	0.85	0.94	0.91	0.81
1e-4	1e-1	0.91	0.94	0.88	0.92	0.88	0.92	0.91	0.87
1e-4	1e-2	0.92	0.91	0.83	0.94	0.86	0.86	0.92	0.93
1e-4	1e-3	0.89	0.88	0.85	0.93	0.84	0.94	0.95	0.95
1e-4	1e-4	0.90	0.92	0.91	0.92	0.94	0.92	0.88	0.85
1e-4	1e-5	0.89	0.91	0.88	0.93	0.92	0.93	0.91	0.87
1e-5	0	0.84	0.94	0.91	0.93	0.93	0.88	0.91	0.91
1e-5	1e-1	0.88	0.95	0.85	0.95	0.85	0.89	0.85	0.88
1e-5	1e-3	0.85	0.90	0.91	0.88	0.89	0.85	0.86	0.86
1e-5	1e-4	0.92	0.82	0.95	0.87	0.90	0.85	0.89	0.91
1e-5	1e-5	0.90	0.91	0.89	0.89	0.91	0.91	0.91	0.87
RF		0.85	0.93	0.95	0.91	0.83	0.90	0.95	0.91
SVM		0.88	0.92	0.95	0.91	0.93	0.92	0.95	0.94
XGBoost		0.82	0.90	0.92	0.65	0.84	0.88	0.91	0.92
ELM		0.65	0.67	0.66	0.60	0.62	0.75	0.64	0.77
GBDT		0.74	0.81	0.77	0.56	0.73	0.90	0.80	0.92
ADLER1		0.86	0.88	0.89	0.83	0.88	0.91	0.92	0.89
DeepCC		0.89	0.94	0.92	0.91	0.89	0.91	0.93	0.86
DeepForest		0.91	0.94	0.93	0.86	0.92	0.93	0.94	0.93
Inception_Res		0.78	0.74	0.77	0.55	0.63	0.80	0.75	0.70
CMSclassifier		0.85	0.91	0.90	0.87	0.90	0.90	0.96	0.90

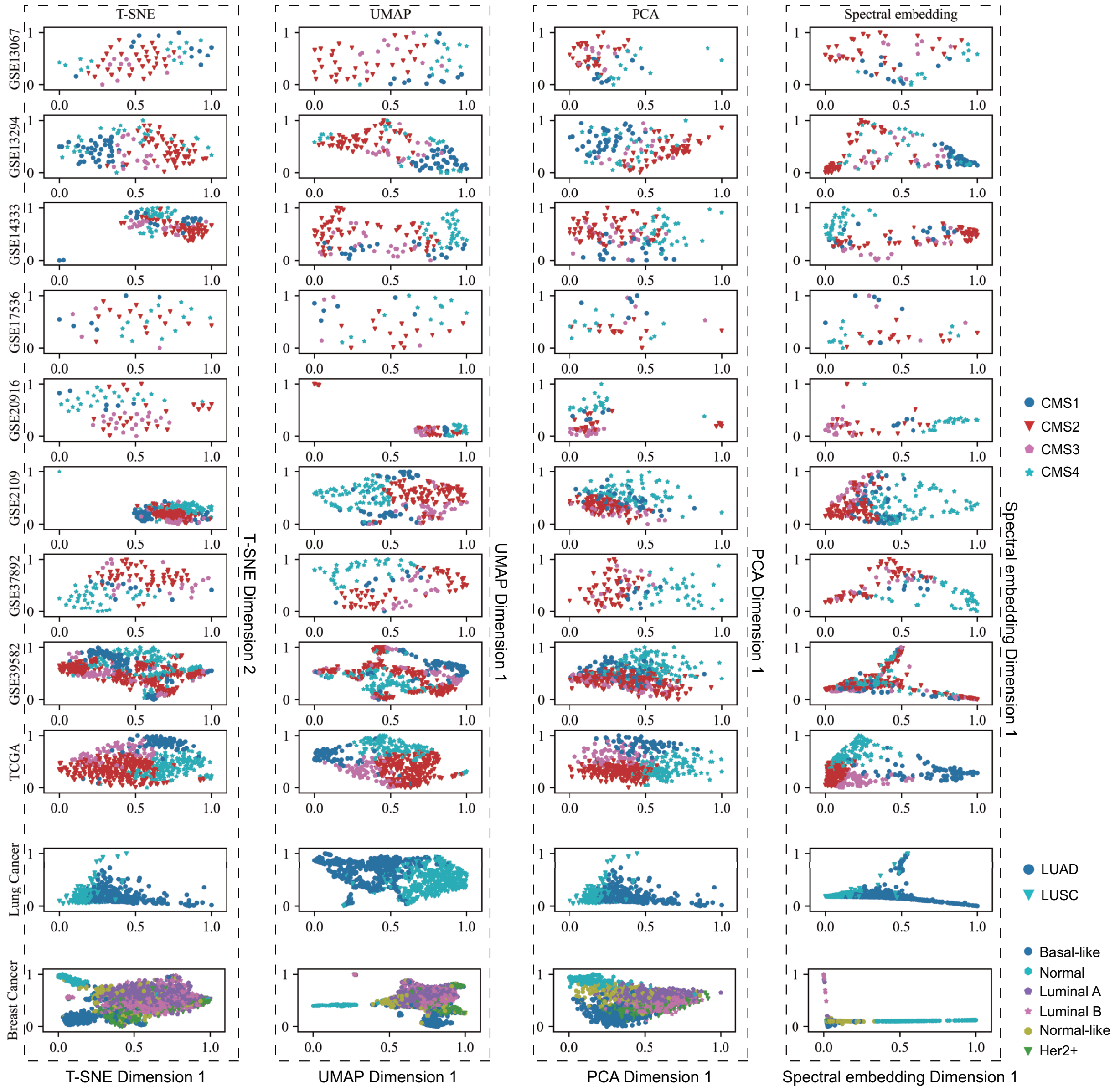

Supplementary Fig.S1: The two-dimensional projection across multiple cancer types using T-SNE, UMAP, PCA and Spectral embedding, respectively. Each type marker represents a cancer subtype.

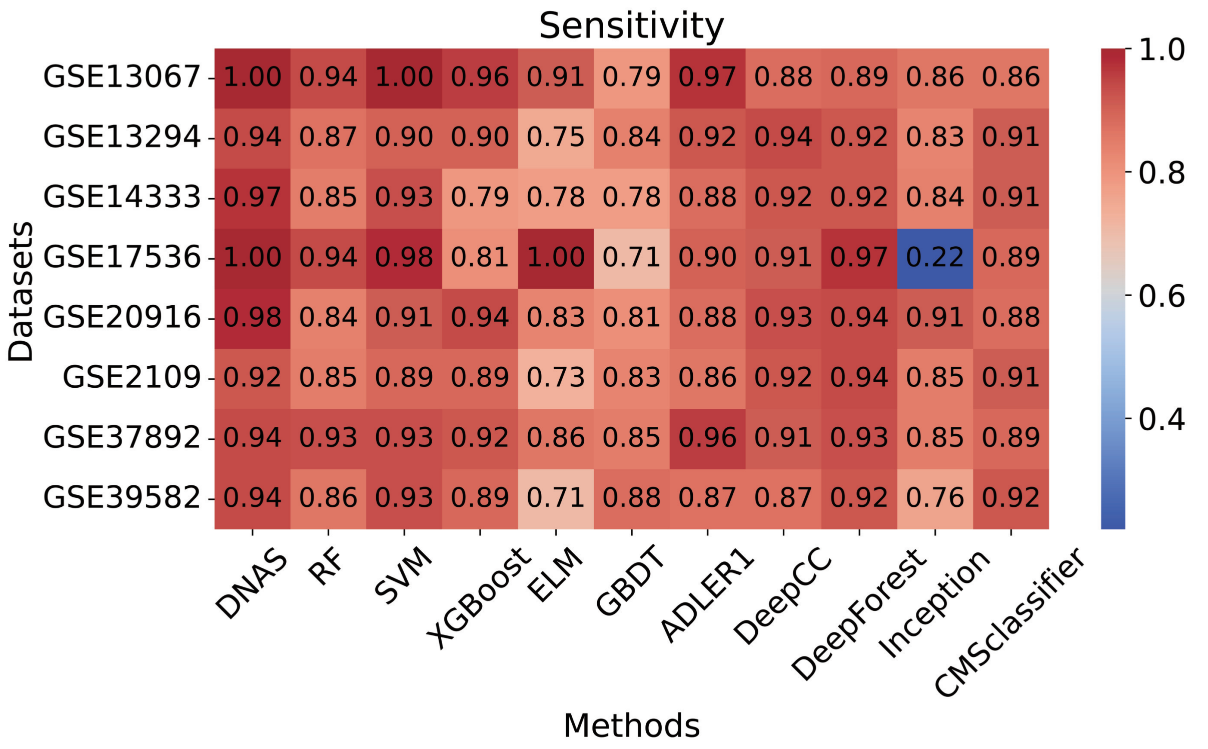

Supplementary Fig.S2: Sensitivity of each method in each dataset.

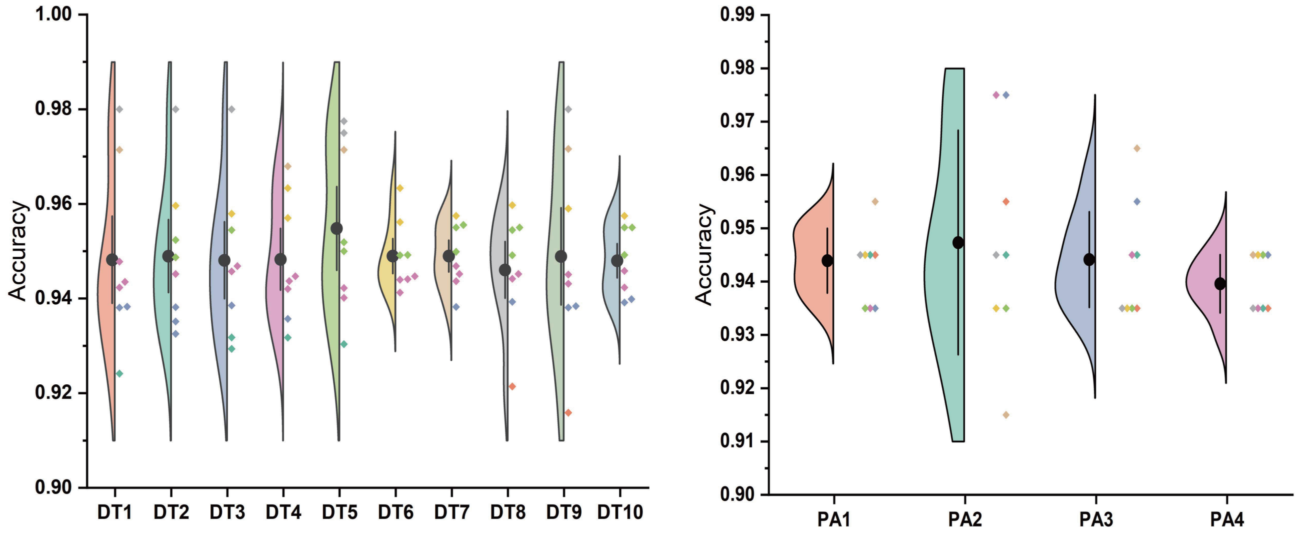

Supplementary Fig.S3: Parameters analysis of DNAS.

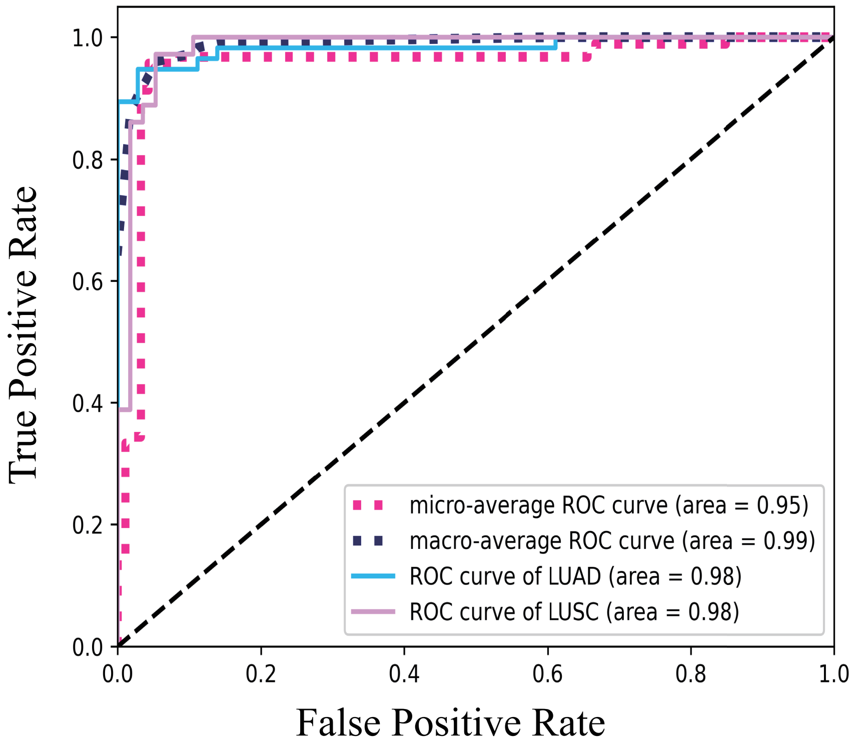

Supplementary Fig.S4: The area under the receiver operating characteristics of DNAS on the test set.

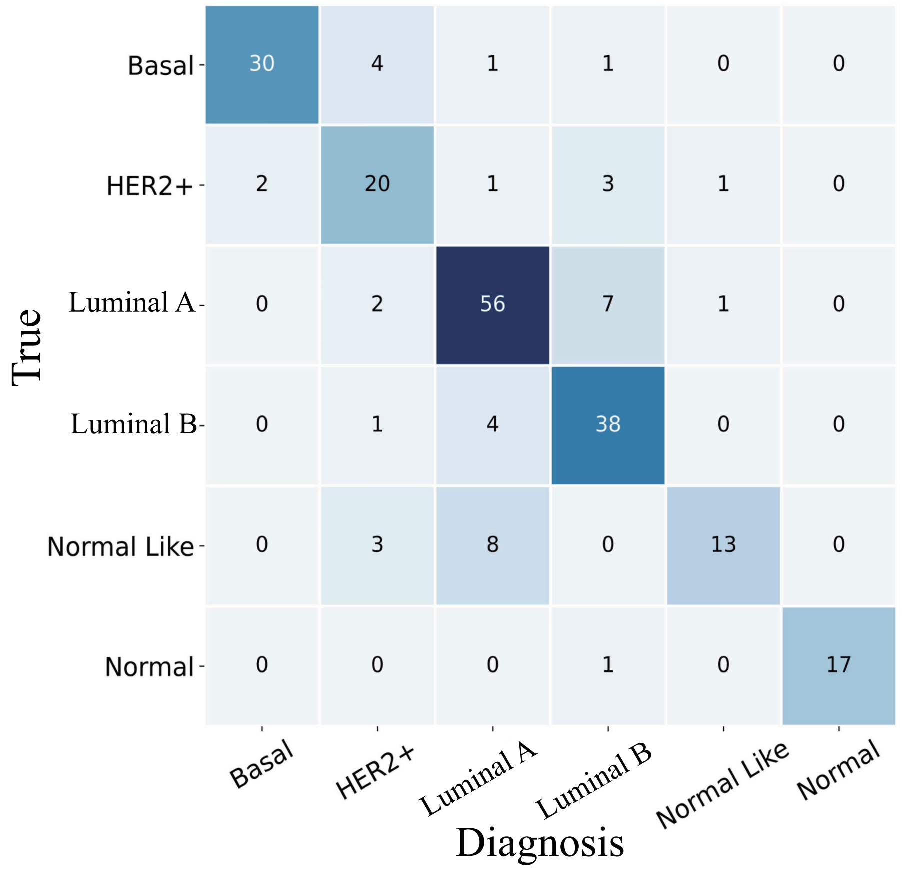

Supplementary Fig.S5: The confusion matrix of DNAS on the test set of breast cancer.

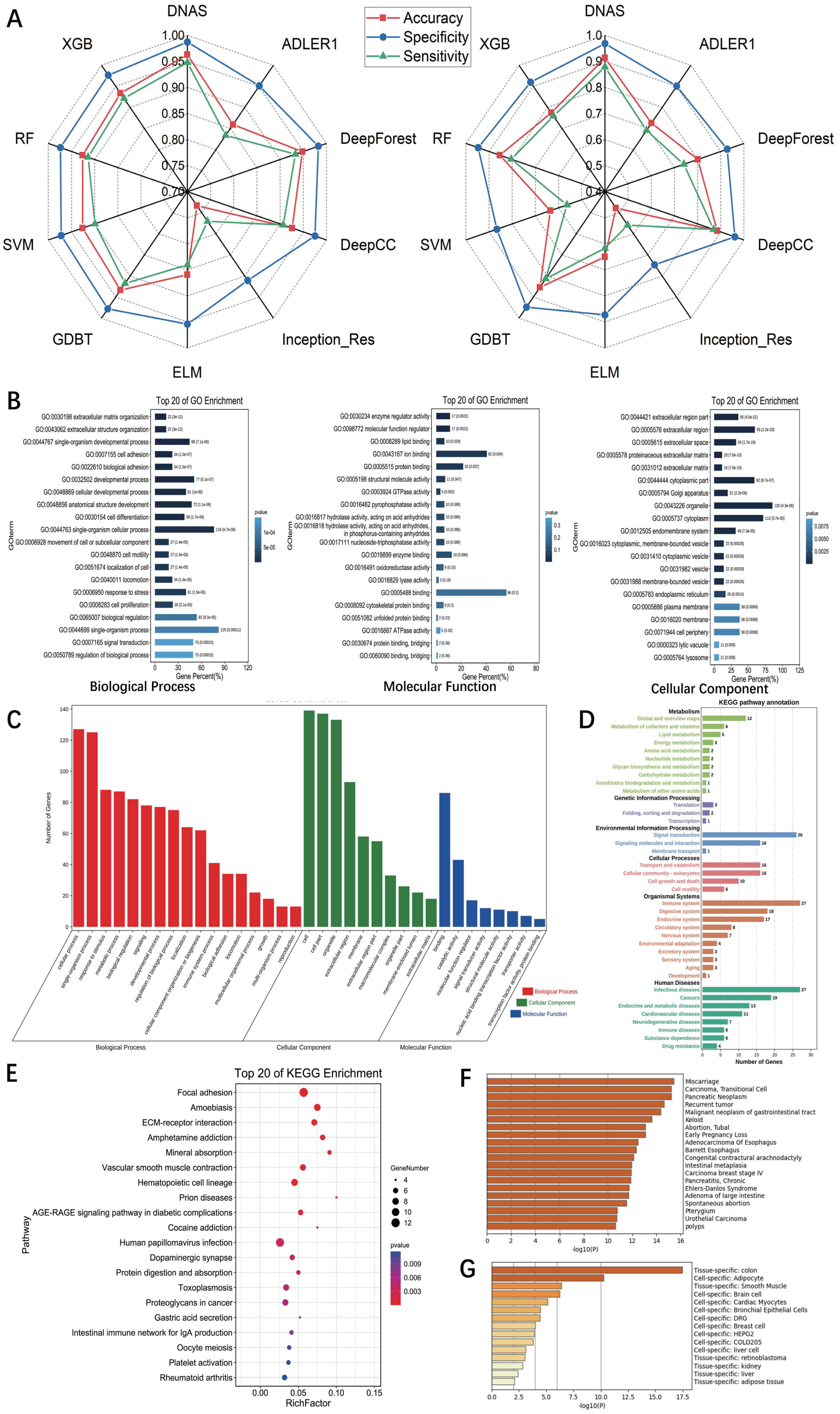

Supplementary Fig.S6: (A) The comparison analysis of external cohort. (B) The top 20 categories of GO enrichment ordered by p-value in terms of biological processes, molecular function, and cellular component. (C) Dispersion of gene associated GO in three different GO enrichment functions. (D) KEGG analysis. The Y-axis lists the distribution of KEGG for seven different pathways including Metabolism, Genetic Information Processing, Environmental Information Processing, Cellular Processes, Organismal Systems, and Human Diseases. (E) The Top 20 KEGG pathways, ordered by p-value. (F) The summary of the enrichment analysis in DisGeNET. (G) The summary of the enrichment analysis in PaGenBase.

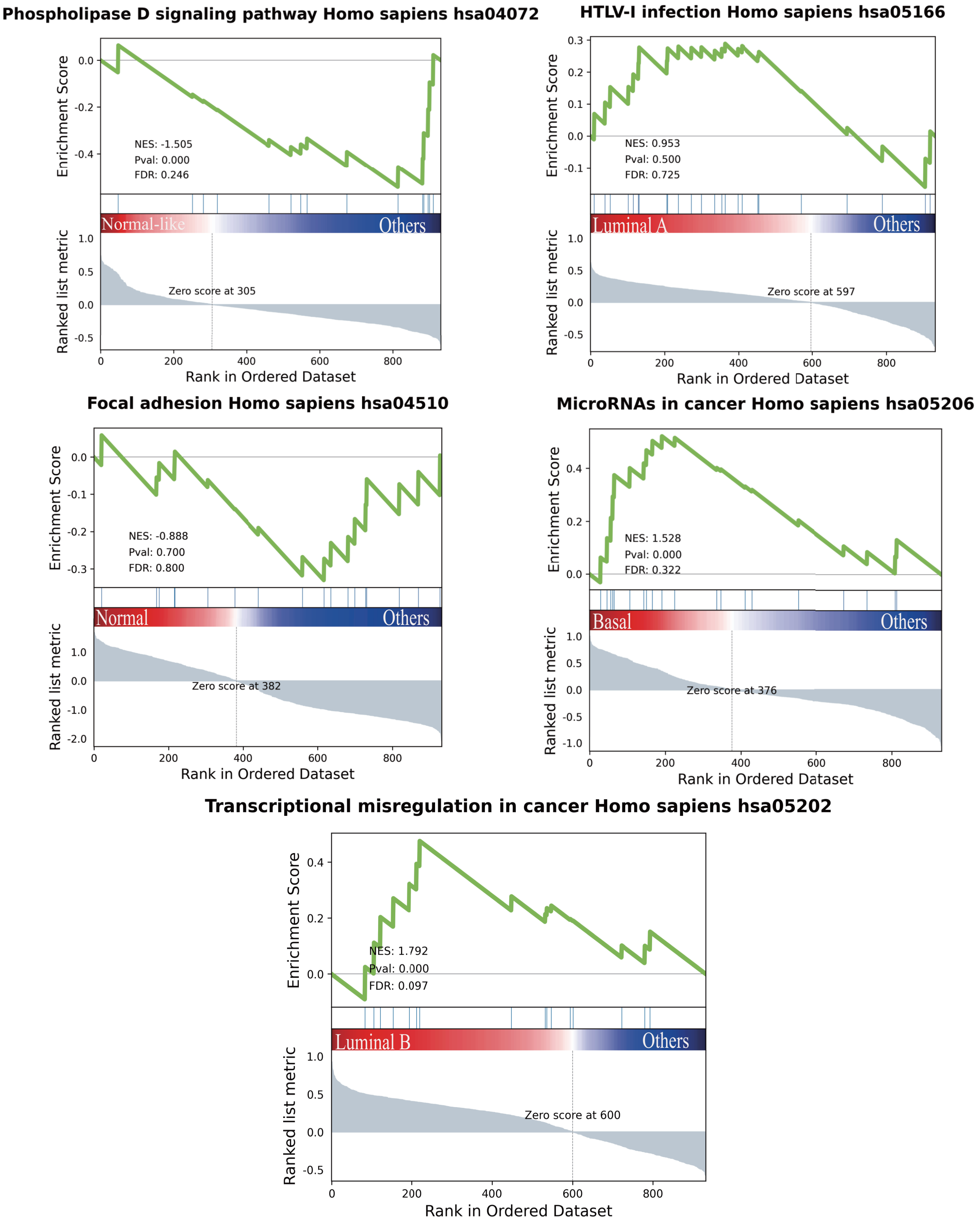

Supplementary Fig.S7: GSEA plot illustrating a representative pathway dysregulated in Normal-like, Luminal A, Normal, Basal and Luminal B identified.

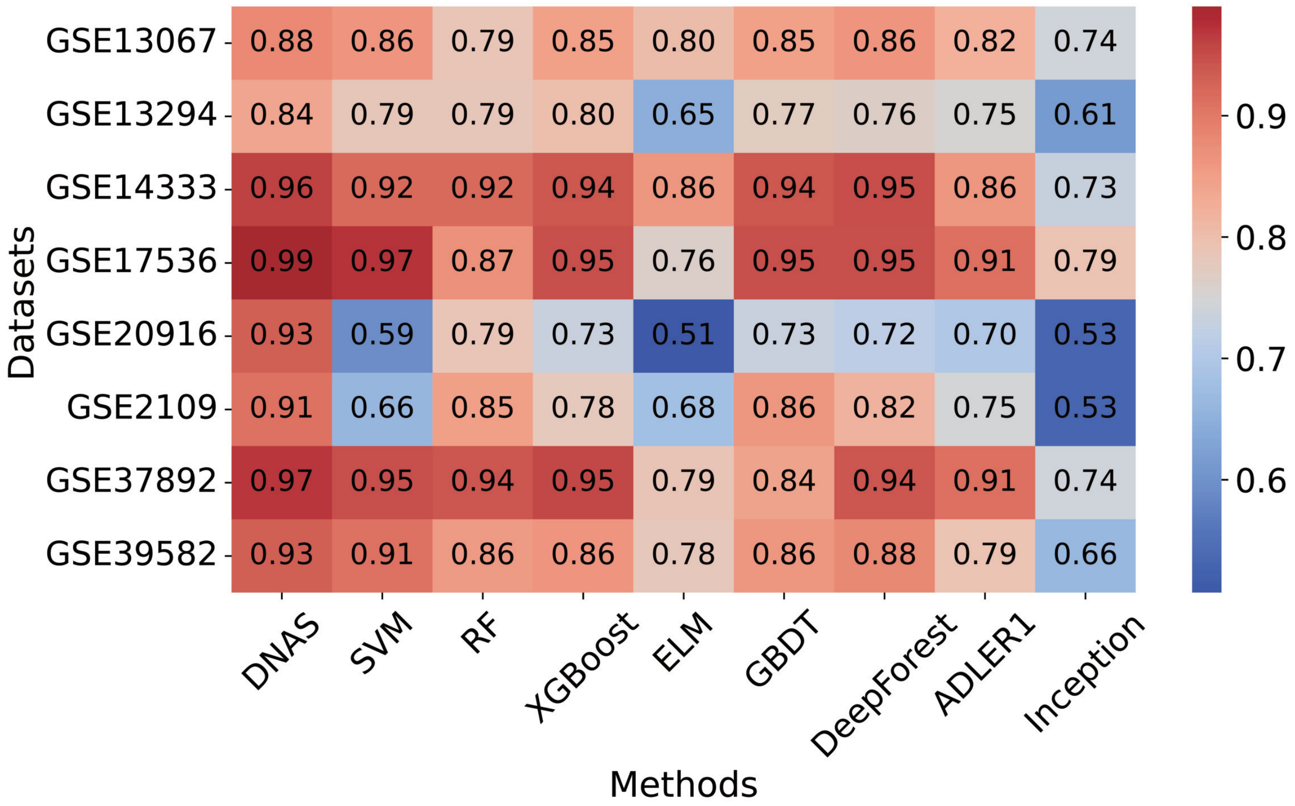

Supplementary Fig.S8: The external cohort comparison experiments across multiple gene expression data. Each row is an independent test set, and the remaining combined data is treated as the training set.

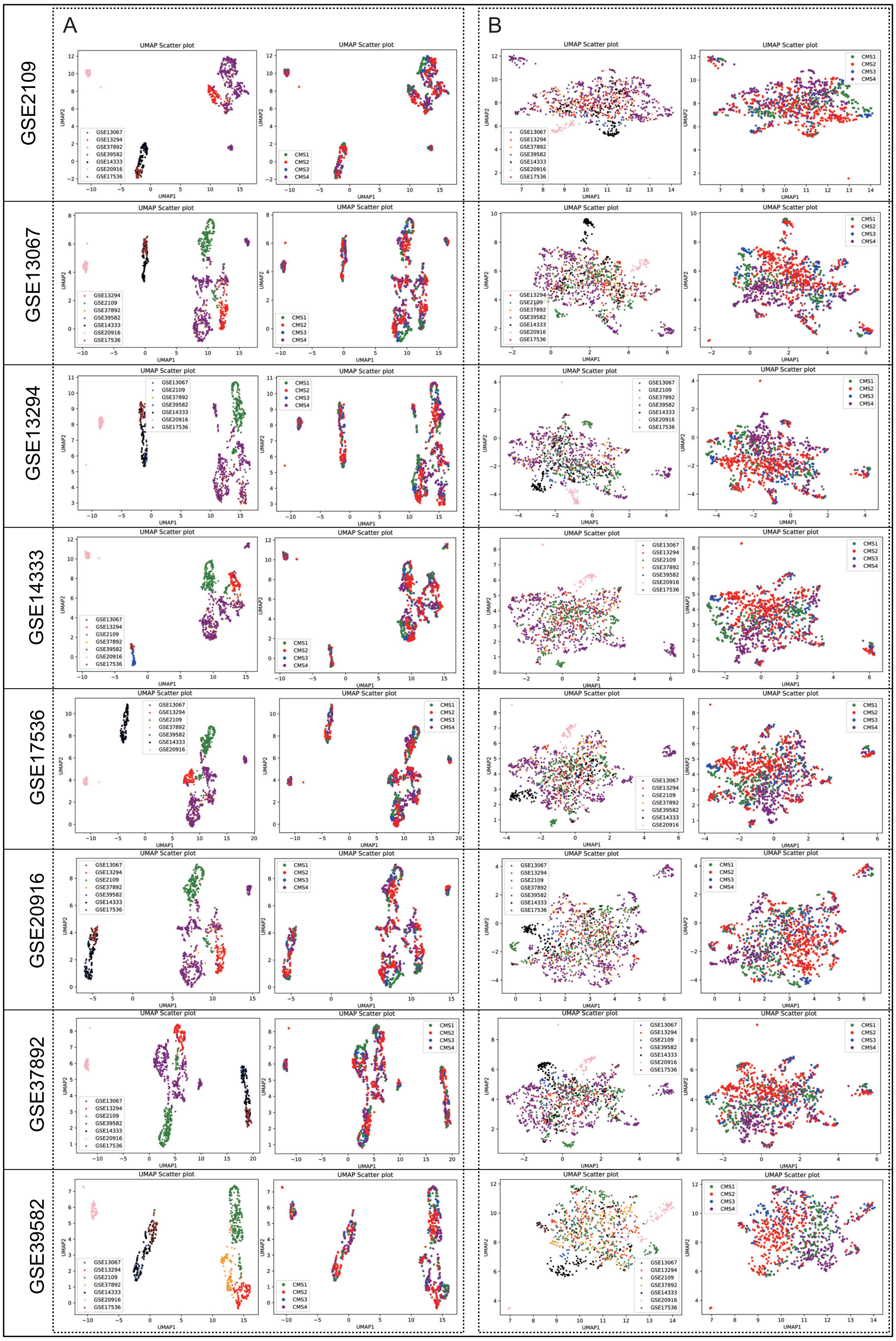

Supplementary Fig.S9: Distribution of data and corresponding labels excluding a specific dataset. (A) Distribution of data and corresponding labels before batch effect removal. (B) Distribution of data and corresponding labels after batch effect removal.

Parameter Settings

We evaluated the performance of DNAS on colorectal cancer subtypes diagnosis. We trained each model on the training data and then evaluated the test data. In DNAS, we chose different hyperparameters from different pools to assemble a single ADLER architecture, including the learning rate pool (1e-1, 1e-2, 1e-3, 1e-4, 1e-5), the optimizer pool (Adam, SGD, Adadelta, Adagrad, RMSprop), the activate function pool (Relu, Softplus, Softsign, Tanh, Selu, Elu), the number of neurons pool (8, 32, 64, 128, 256, 512, 768, 1024), the batch size pool (32, 64, 128, 256), the dropout rate pool (0, 0.1, 0.2, 0.3, 0.4, 0.5), the batch normalization pool (0, 1), the epoch pool (300, 400, 500, 1000, 2000), the L1 ratio pool (0, 1e-1, 1e-2, 1e-3, 1e-4, 1e-5), the L2 ratio pool (0, 1e-1, 1e-2, 1e-3, 1e-4, 1e-5), and the number of fully connected layers pool (1, 2, 3, 4, 5, 6, 7). For the ACO evolution step, the population size N was set to 10, and the maximum iteration to 10. For the dynamic weighted hyperparameter model, the number of decision trees was set to 1000 under the Python scikit-learn package.

Genomic Interpretability

In the previous section, we conduct several experiments that demonstrate the promising performance of DNAS. Next, we investigated the biological significance of DNAS for the external cohort in our study. Firstly, we identified the top 200 genes with the largest weight variances in the first representation layer. In principle, these genes are a "sufficient and necessary" set to represent the model's inputs. After that, multiple enrichment analyses were conducted to elucidate the biological functions.

Supplementary Fig.S6B summarizes the top 20 GO enrichment terms ordered by p-value. In total, we obtained 1945 enriched gene ontology (GO) terms, including 1040 enriched GO biological processes, 714 cellular components, and 191 molecular functions. For biological process, the top five enriched GO terms were extracellular matrix organization (GO:0030198), extracellular structure organization (GO:0043062), single-organism developmental process (GO:0044767), cell adhesion (GO:0007155), and biological adhesion (GO:0022610). Meanwhile, the top five enriched GO cellular component terms were extracellular region part (GO:0044421), extracellular region (GO:0005576), extracellular space (GO:0005615), proteinaceous extracellular matrix (GO:0005578), extracellular matrix (GO:0031012). We found that seven out of the ten identified enriched biological processes and cellular components were associated with the extracellular, which plays an important role in the colorectal microenvironment. The top five enriched GO molecular functions were enzyme regulator activity (GO:0030234), molecular function regulator (GO:0098772), lipid binding (GO:0008289), ion binding (GO:0043167), protein binding (GO:0005515). The distribution of the related gene ontology terms is illustrated in Supplementary Fig.S6C. Multiple terms were assigned to the same genes such as: for the biological process, most genes were

assigned to "cellular process" (15480, 84.63%) and "single-organism process" (12560, 68.67%); for the cellular component, most genes were assigned to "intracellular" (14878, 75.55%) and "intracellular part" (14327, 72.75%); for the molecular function, most genes were assigned to "binding" (10392, 52.48%) and "catalytic activity" (5311, 26.82%).

In addition, the top 200 genes were used in the Kyoto Encyclopedia of Genes and Genomes pathways (KEGG) analysis. Supplementary Fig.S6D summarizes the results of the KEGG-enriched terms. With this, the 8514 unigenes were classified into 215 pathways. Most of them were "Metabolic pathways" (1416, 16.63%). In particular, "Intestinal immune network for IgA production (ko:04672)" (122, 1.43%) with p-value 0.007696 and "PI3K-Akt signaling pathway (ko:04151)" (114, 1.34%) with p-value 0.000994 play a significant role in colorectal cancer. Supplementary Fig.S6E depicts the top 20 KEGG terms ordered by p-value. Thus, the results of KEGG suggested that DNAS could capture the pathological causes of CRC. In addition, we investigated the diseases and tissues associated with the selected genes as shown in Supplementary Fig.S6F and Supplementary Fig.S6G, visualized using DisGeNET and PaGenBase, respectively. From Supplementary Fig.S6F, we find that the top 200 genes are associated with many types of cancer. In particular, adenoma of large intestine, intestinal metaplasia, malignant neoplasm of gastrointestinal tract, and pancreatic neoplasm have a complex association with colorectal cancer. We also discover that the selected genes are highly correlated with the colon as depicted in Supplementary Fig.S6G. Therefore, we can conclude that the DNAS model shows biological significance for colorectal cancer in the external cohort.
